# Supplementary figures and images for: A new method for identifying the acute respiratory distress syndrome disease based on noninvasive physiological parameters
Source: PLoS One. 2020 Feb 5;15(2):e0226962. doi: 10.1371/journal.pone.0226962 (PMC7001976; doi:10.1371/journal.pone.0226962)

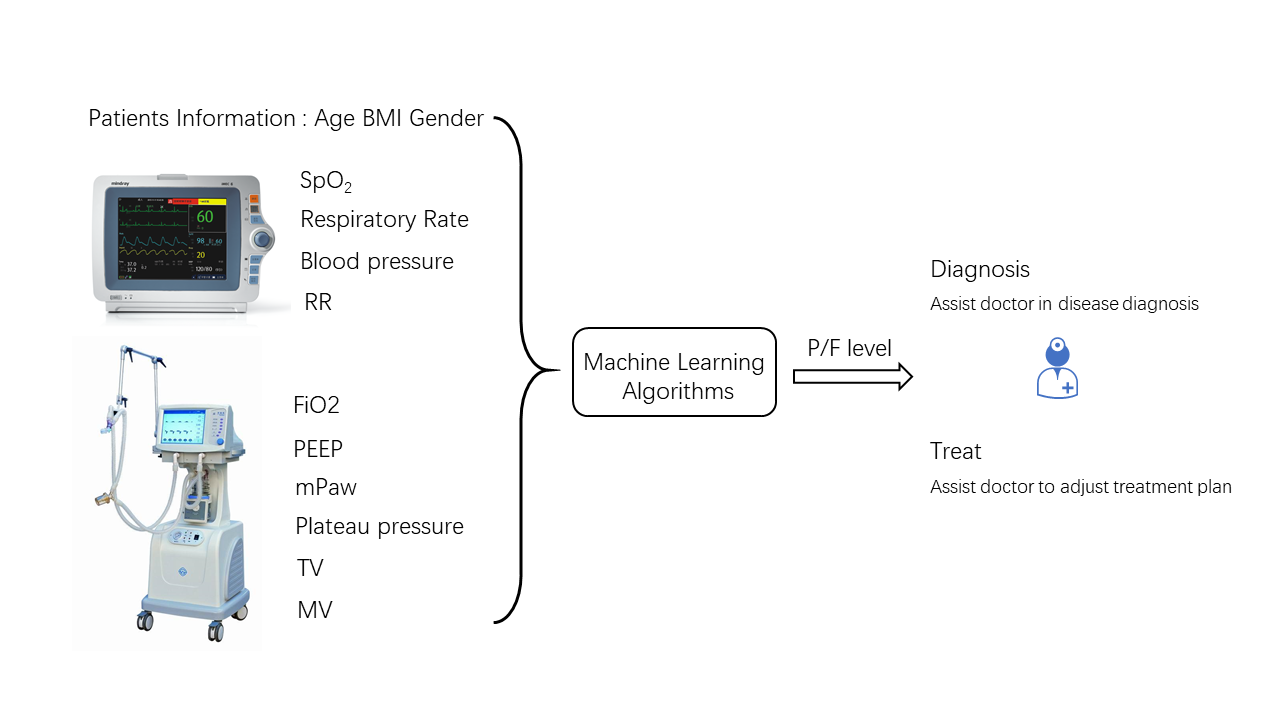

Supplement: S1 Fig — The algorithm continuously monitors the patient's oxygenation level using basic patient information, ventilator parameters, and monitoring parameters to help the doctor diagnose whether the patient has ARDS and adjust the treatment plan for ARDS patients. (TIF) [file pone.0226962.s001.tif]
